# Dataset concerning the hourly conversion factors for the cumulative energy demand and its non-renewable part, and hourly GHG emission factors of the Swiss mix during a one year period (2015–2016)

**DOI:** 10.1016/j.dib.2018.10.090

**Published:** 2018-10-26

**Authors:** Didier Vuarnoz, Thomas Jusselme

**Affiliations:** Ecole Polytechnique Fédérale de Lausanne (EPFL), Building 2050 research group, Passage du Cardinal 13B, CH-1700 Fribourg, Switzerland

## Abstract

The data presented in this article are related to the research article entitled “Temporal variations in the primary energy use and greenhouse gas emissions of the electricity provided to the Swiss grid” Vuarnoz and Jusselme (2018). The provided data are the hourly CO_2-eq_ emission factors, and the hourly conversion factors for the cumulative energy demand and its non-renewable part for the Swiss electricity mix over one year. These data have been assessed on the basis of an inventory of the technology used for electricity generation and an attributional life-cycle approach. The presented data are necessary for life-cycle assessment of all processes and products using electricity in Switzerland. They serve also as a sustainable benchmark when implementing renewable energy systems and energy storage.

**Specifications table**

**Table:** 

Subject area	*Electricity mix*
More specific subject area	*Life-cycle assessment of electricity, i.e. hourly emission factor, hourly conversion factors for the cumulative energy demand and its non-renewable part*
Type of data	*Excel file*
How data was acquired	*Application of the Input-Output assessment model described in**[1]**for the analyzed period (28/01/2015–27/01/2016) with hourly input data from:*
	– *for the inventory of technology involved in electricity generation*: • *EEX* *[2]* *for Switzerland, Germany and Austria* • *RTE* *[3]* *for France* – *for the amount of electricity imports*: • *Swissgrid* *[4]* *for Switzerland* • Itten et al. [5] – *for the technology-specific conversion factors*: • *KBOB database* [6]
Data format	*Raw*
Experimental factors	*The life-cycle assessment is performed with “cradle-to-grave” system boundaries.*
	*Transport and distribution losses are included in the assessment.*
Experimental features	*The reference time is GMT+1*
	*The data are given for a functional unit of 1 kW h of electricity*
Data source location	*Switzerland*
Data accessibility	*Data are with this article*
*Related research article*	D.Vuarnoz, T.Jusselme, Temporal variations in the primary energy use and greenhouse gas emissions of the electricity provided to the Swiss grid. Energy 161: 573–582 [1].

**Value of the data**

• The dataset can be directly used to compute life-cycle assessment (LCA) of processes and products using electricity.
• The dataset can serve to develop time-dependent strategies of electricity use for primary energy optimization and greenhouse gases emission mitigation.
• The dataset can be compared with the dataset of electricity mixes from different regions/countries.
• The dataset can serve as a benchmark, e.g. for the same national grid mix during other period of time, and for a sustainable implementation of renewable energy system and energy storages.

## 1 Data

The data provided within this article consist of hourly conversion factors for the cumulative energy demand (CED) and its non-renewable part (CEDnr), both in (MJ_oil-eq_/kW h), as well as the CO_2-eq_ emission factors (GWP) in (kg CO_2−eq_/kW h) of the electricity provided by the Swiss mix during a one-year period (28/01/2015–27/01/2015). See the .xlsx file.

## 2 Experimental design, materials, and methods

The methodology used to generate the dataset presented in this article is detailed in Ref. [1]. The method consider an input-output model. Any pre-treatment of the input data has been performed and no filter has been applied to the obtained dataset. Original input data used for the assessment originate from different sources and consist of hourly inventories of domestic productions, hourly electricity imports/exports and technology-specific conversion factors. For each domestic production, data from the inventory are (1) the energy generation per hour (kW h/h) and (2) the types of technology used. Regarding the domestic productions, the inventory of the technologies involved during each hour has been provided by [2] for Switzerland, Germany and Austria. As for the inventory of France, we use the data from [3]. The technology-specific conversion factors used for the assessment are those from the KBOB database [4]. With regard to the electricity imports, hourly values of the Swiss imports have been provided by Itten et al., (2014) [5]. French, Austrian, and German imports have been assumed to be constant over one year, and corresponding to the mean annual values given in Ref. [6].
